# When Hematologic Response Does Not Translate to Cardiac Recovery in AL Amyloidosis

**DOI:** 10.1016/j.jaccas.2026.109011

**Published:** 2026-09-23

**Authors:** Pragyat Futela, Poornima Vinod, Bala Pushparaji, Maxine Nelson, Khalil Murad, Ashish Aneja, Kathir Balakumaran

**Affiliations:** a Department of Internal Medicine, MetroHealth Medical Center, Cleveland, Ohio, USA; b Department of Cardiovascular Medicine, MetroHealth Medical Center, Cleveland, Ohio, USA

**Keywords:** cardiac amyloidosis, discordant hematologic and cardiac response, light-chain amyloidosis, right ventricular dysfunction

## Abstract

**Background:**

Cardiac light-chain (AL) amyloidosis carries high early mortality due to rapid hemodynamic deterioration. Hematologic response does not always translate into cardiac recovery, particularly in advanced disease.

**Case Summary:**

A 66-year-old woman presented with dyspnea and chronic edema. Electrocardiography showed low QRS voltage, and echocardiography revealed severe concentric thickening with apical sparing on strain imaging. Free λ light chains were markedly elevated, and bone marrow biopsy confirmed plasma cell myeloma. Cardiac magnetic resonance imaging supported infiltrative cardiomyopathy. Daratumumab-based chemotherapy resulted in substantial reduction in free λ levels. Despite hematologic response, she remained hypotensive with persistent right ventricular dysfunction and intolerant to guideline-directed therapy. Five months after presentation, she died suddenly at home.

**Discussion:**

This case illustrates the dissociation between biochemical improvement and cardiac stabilization in advanced AL amyloidosis and highlights the persistent risk of hemodynamic collapse and sudden death.

**Take-Home Message:**

Early hematologic response does not ensure cardiac stabilization in advanced cardiac AL amyloidosis.

## Graphical Abstract

**Figure:**
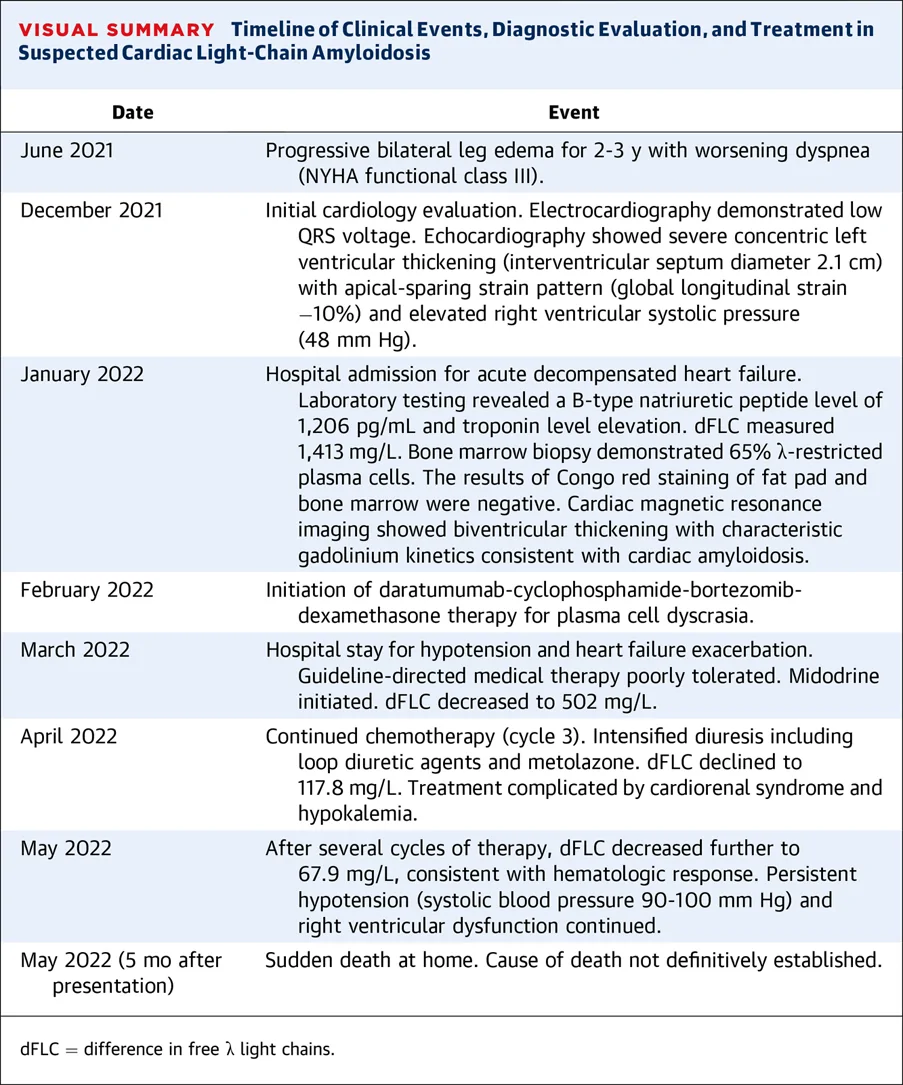

## History

A 66-year-old African-American woman presented with progressive dyspnea on exertion and bilateral lower extremity edema for the past several years, worsening over months. She reported orthopnea and reduced exercise tolerance consistent with NYHA functional class III symptoms. She denied a history of chest pain, palpitations, presyncope, or syncope.

Physical examination revealed elevated jugular venous pressure, bilateral basal crackles, and 3+ bilateral pitting edema with chronic skin changes in the lower extremities. Cardiac examination demonstrated regular rate and rhythm with normal S_1_ and S_2_. The extremities were warm with normal capillary refill.

## Past Medical History

Her history was notable for chronic obstructive pulmonary disease without prior cardiac disease. A prior nuclear cardiac stress test result was negative. Family history was significant for sudden cardiac death in a sibling, reportedly described as having a “thick heart.”

## Differential Diagnosis

The differential diagnosis for concentric left ventricular thickening with low-voltage electrocardiography in an elderly woman presenting with congestive symptoms included infiltrative cardiomyopathy (light-chain [AL] amyloidosis and transthyretin amyloidosis), hypertensive heart disease, and hypertrophic cardiomyopathy.

## Investigations

**Figure 1:**
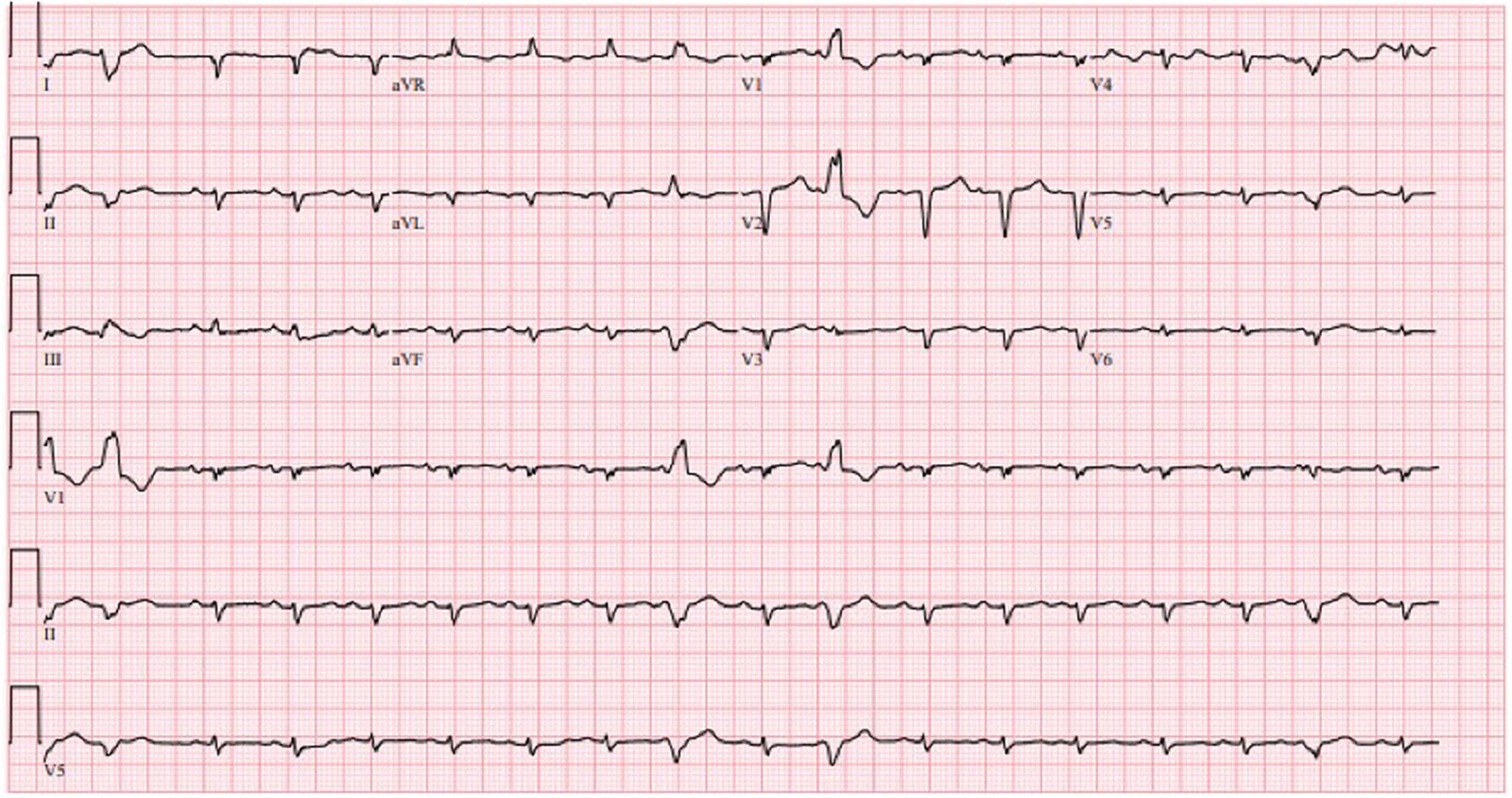
Electrocardiogram Showing Low QRS Voltage

**Figure 2:**
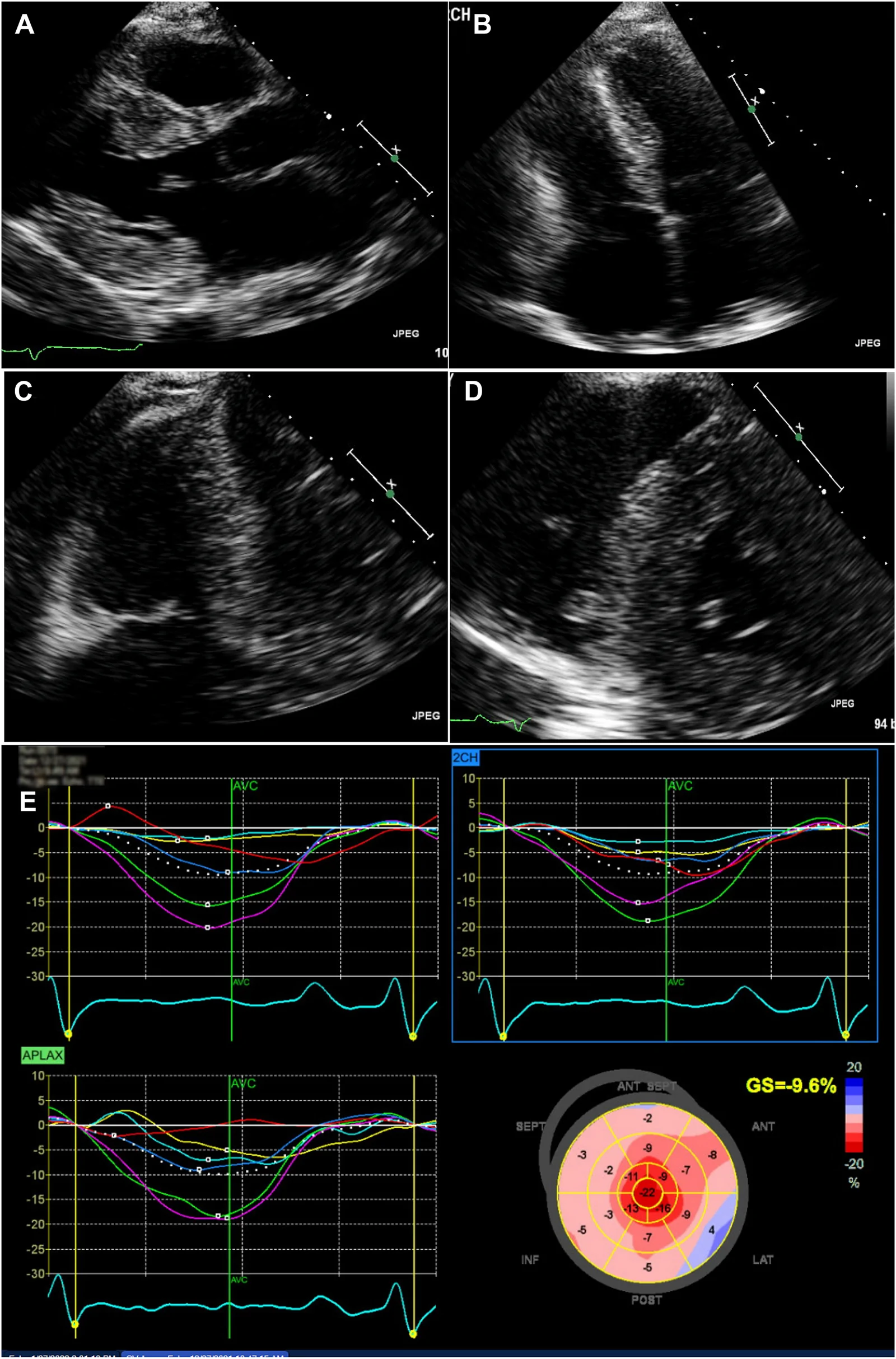
Two-Dimensional Echocardiogram (A) Parasternal long-axis view showing concentric left ventricular thickening. (B) Apical 4-chamber view showing biatrial enlargement. (C) Right ventricle–focused view showing mild right ventricular dilation. (D) Parasternal short-axis view showing left ventricular thickening. (E) Strain imaging showing apical sparing.

Cardiac biomarkers indicated myocardial injury and ventricular wall stress with a B-type natriuretic peptide (BNP) level of 1,206 pg/mL and a troponin I level of 0.339 ng/mL. Electrocardiography demonstrated low QRS voltage, left posterior fascicular block, and ventricular ectopy (Figure 1). Transthoracic echocardiography revealed severe concentric left ventricular thickening (interventricular septum diameter 2.1 cm), preserved ejection fraction (55% to 60%), apical-sparing strain pattern (global longitudinal strain −10%), severe left atrial enlargement (left atrial volume index ∼41 mL/m^2^), and mild right atrial enlargement (Figure 2). Doppler parameters demonstrated impaired relaxation with reduced mitral annular velocities (septal e′ 0.04 m/s, lateral e′ 0.06 m/s) and elevated right ventricular systolic pressure (48 mm Hg). Discordant low electrocardiographic voltage despite marked left ventricular wall thickening with apical sparing raised a strong suspicion for infiltrative cardiomyopathy.

**Figure 3:**
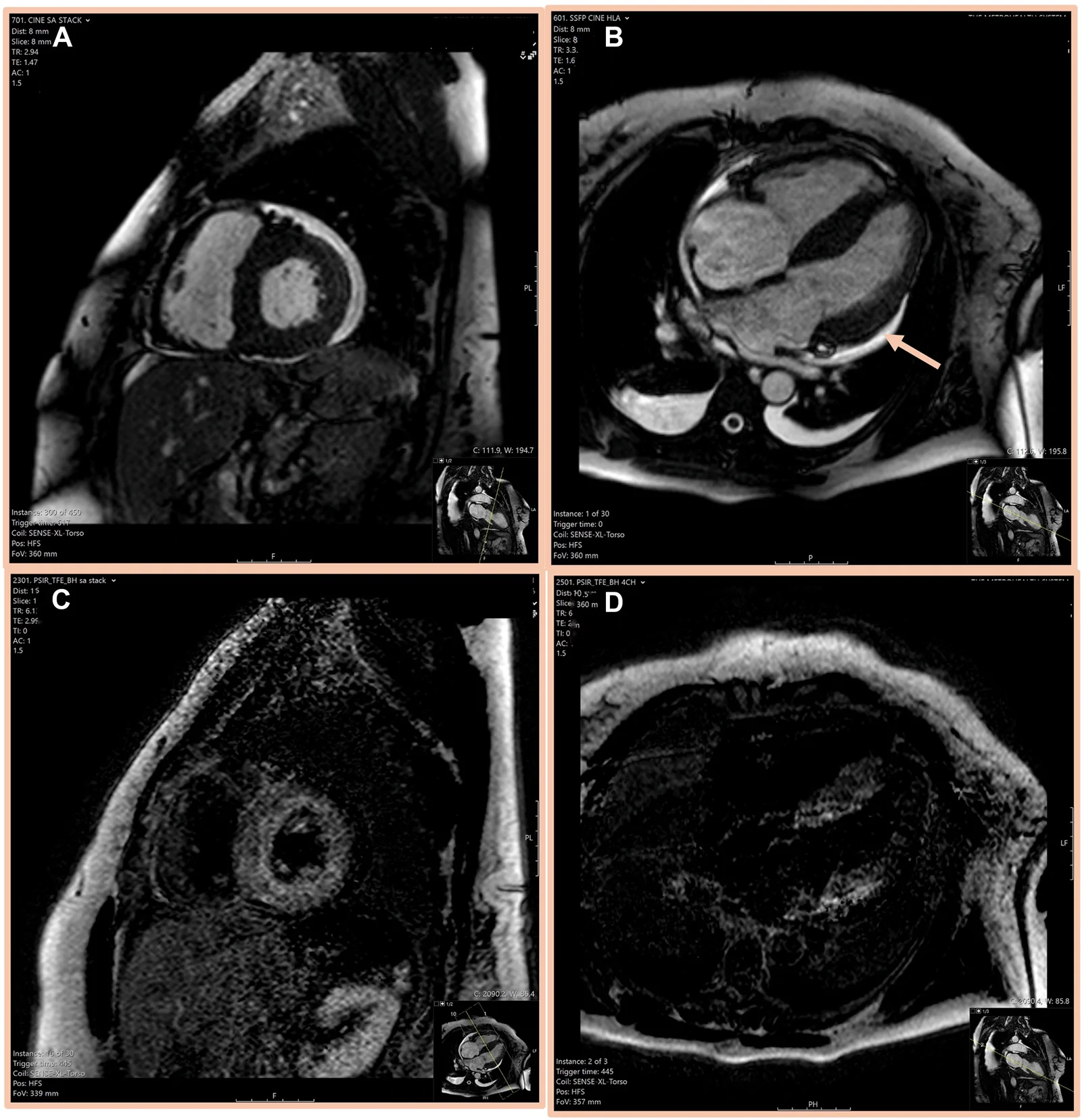
Cardiac Magnetic Resonance Image (A) Short-axis basal left ventricular steady-state free precession cine image in end diastole. (B) Four-chamber steady-state free precession cine image in end diastole, demonstrating biventricular wall thickening and biatrial enlargement. (C and D) Phase-sensitive inversion recovery postgadolinium images with gadolinium uptake in the myocardium, particularly the basal and mid–left ventricular segments (image D), showing absence of gadolinium in the blood pool at an inversion time of 320 ms after a double dose of gadolinium contrast. Images were acquired approximately 10 to 12 minutes after administering gadolinium contrast. These findings are characteristic of cardiac amyloidosis.

Serum free light-chain (FLC) analysis revealed markedly elevated λ FLCs (1,419 mg/L) with suppressed κ levels (difference in FLCs [dFLC] 1,413 mg/L). Bone marrow biopsy demonstrated 65% λ-restricted plasma cells consistent with plasma cell myeloma, λ restricted. fluorescence in situ hybridization studies identified a high-risk cytogenetic profile including t(14;16) immunoglobulin heavy chain/MAF fusion, gain 1q21, and del 13q14. The results of Congo red staining of bone marrow and abdominal fat pad were negative. Technetium-99m pyrophosphate scintigraphy demonstrated no myocardial uptake. Cardiac magnetic resonance (CMR) imaging revealed concentric biventricular thickening, left ventricular ejection fraction 42%, right ventricular ejection fraction 25%. Phase-sensitive inversion recovery postgadolinium imaging revealed myocardial gadolinium uptake in the basal and mid–left ventricular segments with absence of blood pool gadolinium at an inversion time of 320 ms, findings highly suggestive of cardiac amyloidosis (Figure 3). Endomyocardial biopsy was deferred given procedural risk in the setting of biventricular dysfunction, and diagnosis was established on the basis of CMR imaging and bone marrow biopsy findings.

## Management

The patient was initiated on daratumumab-cyclophosphamide-bortezomib-dexamethasone (Dara-CyBorD) therapy for plasma cell dyscrasia, and the patient was deemed ineligible for autologous hematopoietic stem cell transplantation evaluation, given advanced cardiac involvement.

Heart failure management focused on volume optimization. Loop diuretic agents were intensified, and spironolactone was initiated. Guideline-directed medical therapy was cautiously introduced with low-dose losartan and metoprolol; however, persistent hypotension limited tolerance, necessitating discontinuation of all agents except loop diuretic agents. Midodrine was subsequently initiated for symptomatic hypotension.

Escalation of diuretic therapy, including short-course metolazone, improved congestion and functional capacity but was complicated by cardiorenal syndrome and hypokalemia, requiring close laboratory monitoring.

## Outcome and Follow-up

**Figure 4:**
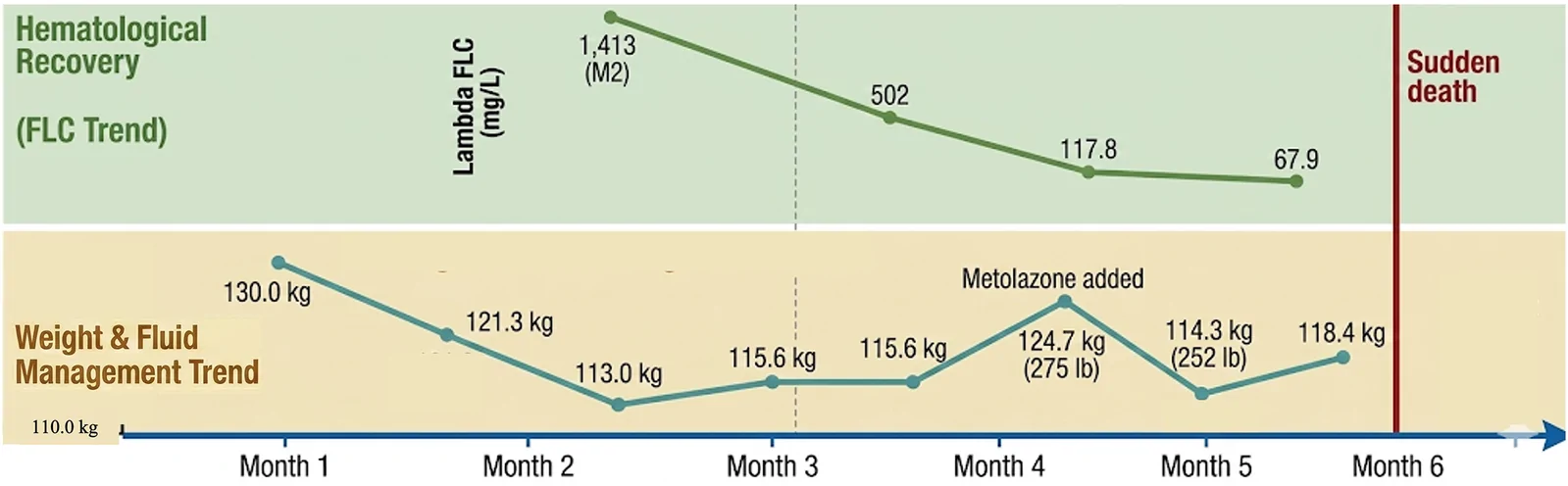
Temporal Relationship Between Difference in Free Light-Chain Reduction and Clinical Status FLC = free light chain.

After several cycles of Dara-CyBorD therapy, dFLC levels declined from 1,413 to 67.9 mg/L over 4 months (Figure 4). Despite hematologic improvement and close multidisciplinary follow-up, she remained persistently hypotensive (systolic blood pressure 90-100 mm Hg), attributed to ongoing right ventricular dysfunction.

Five months after her initial cardiology presentation, she died suddenly at home. The exact cause of death was not established. No syncopal episodes were documented before death.

**VISUAL SUMMARY:** Timeline of Clinical Events, Diagnostic Evaluation, and Treatment in Suspected Cardiac Light-Chain Amyloidosis

Date	Event
June 2021	Progressive bilateral leg edema for 2-3 y with worsening dyspnea (NYHA functional class III).
December 2021	Initial cardiology evaluation. Electrocardiography demonstrated low QRS voltage. Echocardiography showed severe concentric left ventricular thickening (interventricular septum diameter 2.1 cm) with apical-sparing strain pattern (global longitudinal strain −10%) and elevated right ventricular systolic pressure (48 mm Hg).
January 2022	Hospital admission for acute decompensated heart failure. Laboratory testing revealed a B-type natriuretic peptide level of 1,206 pg/mL and troponin level elevation. dFLC measured 1,413 mg/L. Bone marrow biopsy demonstrated 65% λ-restricted plasma cells. The results of Congo red staining of fat pad and bone marrow were negative. Cardiac magnetic resonance imaging showed biventricular thickening with characteristic gadolinium kinetics consistent with cardiac amyloidosis.
February 2022	Initiation of daratumumab-cyclophosphamide-bortezomib-dexamethasone therapy for plasma cell dyscrasia.
March 2022	Hospital stay for hypotension and heart failure exacerbation. Guideline-directed medical therapy poorly tolerated. Midodrine initiated. dFLC decreased to 502 mg/L.
April 2022	Continued chemotherapy (cycle 3). Intensified diuresis including loop diuretic agents and metolazone. dFLC declined to 117.8 mg/L. Treatment complicated by cardiorenal syndrome and hypokalemia.
May 2022	After several cycles of therapy, dFLC decreased further to 67.9 mg/L, consistent with hematologic response. Persistent hypotension (systolic blood pressure 90-100 mm Hg) and right ventricular dysfunction continued.
May 2022 (5 mo after presentation)	Sudden death at home. Cause of death not definitively established.

dFLC = difference in free λ light chains.

## Discussion

This case demonstrates the classic phenotype of cardiac AL amyloidosis, an aggressive plasma cell disorder in which prognosis is largely determined by the severity of cardiac involvement. Although AL amyloidosis typically arises from a small, highly amyloidogenic plasma cell clone (<10% marrow involvement), our patient's 65% λ-restricted plasma cell burden indicated concurrent multiple myeloma, an uncommon overlap occurring in approximately 10% to 15% of AL amyloidosis cases,^1^ associated with higher tumor burden, aggressive cytogenetics, and greater cardiotoxic light-chain production, as reflected by the high-risk t(14;16) fusion.

Contemporary staging systems underscore the prognostic importance of cardiac biomarkers.^2^ In advanced disease, stage IIIb, defined by markedly elevated natriuretic peptides (BNP level ≈700 pg/mL threshold) along with elevated troponin, carries particularly poor outcomes; in a validation study, 43.6% of patients died within 6 months of diagnosis.^3^ The phase 3 ANDROMEDA trial established daratumumab-based therapy (Dara-CyBorD) as the preferred frontline regimen for newly diagnosed AL amyloidosis.^4^ However, patients with the most advanced cardiac disease were under-represented in pivotal trials.

In our patient, transthyretin genetic testing was not pursued given the low clinical probability of concurrent transthyretin amyloidosis, supported by the negative pyrophosphate scan, and markedly elevated λ/κ FLC ratio. Nevertheless, the Val122Ile (p.Val142Ile) TTR variant, present in approximately 3% to 4% of individuals of African-American ancestry, warrants consideration in this context, especially given the sibling's history of sudden cardiac death and a “thick heart.”^5^ Its identification carries significant implications for both the patient and cascade screening of at-risk relatives. Although tissue confirmation of amyloid deposition is generally required, extracardiac biopsy sensitivity in AL amyloidosis ranges from 50% to 80%. In our patient, the diagnosis was supported by characteristic CMR imaging gadolinium kinetics alongside confirmed plasma cell myeloma and markedly elevated dFLC. Clinicians should remain aware that AL deposition disease can rarely mimic this phenotype without true amyloid deposition; however, characteristic CMR imaging gadolinium kinetics argue strongly against light-chain deposition disease and in favor of true fibrillar amyloid deposition.

Cardiac dysfunction in AL amyloidosis results from both extracellular amyloid fibril deposition and direct cardiotoxic effects of circulating light chains. Amyloidogenic light chains induce oxidative stress and reactive oxygen species generation, disrupt calcium handling through Sarco/endoplasmic reticulum Ca²⁺-ATPase 2a dysregulation, impair mitochondrial oxidative phosphorylation, and activate cardiomyocyte apoptosis through pathways including Forkhead box O3a-Beclin-1–mediated autophagy and noncanonical p38α mitogen-activated protein kinase signaling.^6^^,^^7^ These proteotoxic mechanisms help explain why AL amyloidosis often produces disproportionately severe myocardial dysfunction despite relatively modest ventricular thickening, in contrast to transthyretin amyloidosis, where dysfunction more closely parallels amyloid burden.^8^ Suppression of circulating toxic light chains may therefore precede structural regression of myocardial amyloid deposits, and early functional changes, particularly in longitudinal strain, may provide sensitive markers of myocardial involvement before overt structural remodeling becomes apparent.^9^

Our patient illustrates the clinical challenge of advanced cardiac AL amyloidosis despite prompt initiation of contemporary therapy. Although daratumumab-based regimens frequently achieve deep hematologic responses (very good partial response or better in approximately 80% to 90% of patients), organ responses are less frequent and often delayed.^10^ In our case, despite an approximately 95% reduction in circulating FLC, residual levels of 67.9 mg/L exceeded the very good partial response threshold of <40 mg/L, suggesting persistent amyloidogenicity. Persistent hypotension and progressive right ventricular dysfunction reflected advanced cardiac involvement with limited reversibility, highlighting the narrow window for myocardial recovery once severe cardiac injury is established.

Sudden cardiac death in AL amyloidosis most commonly results from electromechanical dissociation rather than ventricular arrhythmia. Our patient harbored several risk factors for sudden death in cardiac AL amyloidosis, including stage IIIb disease, advanced age, prolonged QRS duration, and frequent ventricular ectopy on presenting electrocardiography, high BNP level, right ventricular ejection fraction of 25%, and persistent hypotension despite midodrine.^11^ At the time of chemotherapy initiation, no primary arrhythmic event had occurred to warrant prophylactic defibrillator implantation. In AL amyloidosis, implantable cardioverter-defibrillator candidacy is generally deferred until hematologic response is established and projected survival exceeds 1 year, given the predominance of nonshockable terminal rhythms and high procedural risk in advanced disease. In retrospect, ambulatory rhythm monitoring may have provided additional risk stratification data and informed earlier discussion of device therapy.

In addition, although autologous hematopoietic stem cell transplantation remains a potentially curative strategy in selected patients, eligibility is restricted with strict cardiac biomarker or blood pressure cutoffs because advanced cardiac involvement markedly increases treatment-related mortality. Patients with stage IIIb disease or significant hemodynamic instability frequently fall outside these thresholds.^12^

Overall, this case underscores the aggressive natural history of advanced cardiac AL amyloidosis. Despite guideline-concordant therapy, the combination of advanced biomarker stage, restrictive physiology, and right ventricular involvement may predispose to rapid hemodynamic deterioration, particularly in patients with concurrent myeloma, high-risk cytogenetics, and early electrocardiographic risk markers of sudden death. These observations reinforce the importance of early recognition and intervention before progression to severe cardiac involvement when therapeutic reversibility becomes limited.

## Conclusions

Advanced cardiac AL amyloidosis may demonstrate marked dissociation between hematologic response and cardiac recovery. Persistent hemodynamic fragility can occur despite appropriate plasma cell–directed therapy, contributing to early mortality.

## Funding support and author disclosures

The authors have reported that they have no relationships relevant to the contents of this paper to disclose.

**Take-Home Messages**

• Hematologic response in advanced cardiac light-chain amyloidosis does not necessarily translate into myocardial recovery.
• Patients exhibit marked hemodynamic fragility with intolerance to conventional heart failure therapies, emphasizing the importance of early diagnosis before severe cardiac involvement.
